# Cytotoxic Potential of Betulinic Acid Fatty Esters and Their Liposomal Formulations: Targeting Breast, Colon, and Lung Cancer Cell Lines

**DOI:** 10.3390/molecules29143399

**Published:** 2024-07-19

**Authors:** Andreea Milan, Marius Mioc, Alexandra Mioc, Armand Gogulescu, Gabriel Mardale, Ștefana Avram, Tamara Maksimović, Bogdan Mara, Codruța Șoica

**Affiliations:** 1 Faculty of Pharmacy, Victor Babes University of Medicine and Pharmacy, Eftimie Murgu Square, No. 2, 300041 Timișoara, Romania; andreea.milan@umft.ro (A.M.); marius.mioc@umft.ro (M.M.); alexandra.mioc@umft.ro (A.M.); mardale.gabriel@umft.ro (G.M.); stefana.avram@umft.ro (Ș.A.); tamara.maksimovic@student.umft.ro (T.M.); mara.bogdan@umft.ro (B.M.); codrutasoica@umft.ro (C.Ș.); 2 Research Centre for Pharmaco-Toxicological Evaluation, Victor Babes University of Medicine and Pharmacy, Eftimie Murgu Square, No. 2, 300041 Timișoara, Romania; 3 Faculty of Medicine, Victor Babes University of Medicine and Pharmacy, Eftimie Murgu Square, No. 2, 300041 Timișoara, Romania; 4 Institute of Chemistry Coriolan Drăgulescu, 24 Mihai Viteazu Ave, 300223 Timișoara, Romania

**Keywords:** betulinic acid, fatty esters, liposomes, cytotoxicity, molecular docking, HET-CAM assay

## Abstract

Betulinic acid is a lupane-type pentacyclic triterpene mostly found in birch bark and thoroughly explored for its wide range of pharmacological activities. Despite its impressive biological potential, its low bioavailability has challenged many researchers to develop different formulations for achieving better in vitro and in vivo effects. We previously reported the synthesis of fatty acid esters of betulinic acid using butyric, stearic, and palmitic acids (But-BA, St-BA, and Pal-BA) and included them in surfaced-modified liposomes (But-BA-Lip, St-BA-Lip, Pal-BA-Lip). In the current study, we evaluated the cytotoxic effects of both fatty acid esters and their respective liposomal formulations against MCF-7, HT-29, and NCI-H460 cell line. The cytotoxic assessment of BA derivatives revealed that both the fatty esters and their liposomal formulations acted as cytotoxic agents in a dose- and time-dependent manner. But-BA-Lip exerted stronger cytotoxic effects than the parent compound, BA and its liposomal formulation, and even stronger effects than 5-FU against HT-29 cells (IC_50_ of 30.57 μM) and NCI-H460 cells (IC_50_ of 30.74 μM). BA’s fatty esters and their respective liposomal formulations facilitated apoptosis in cancer cells by inducing nuclear morphological changes and increasing caspase-3/-7 activity. The HET-CAM assay proved that none of the tested compounds induced any irritative effect, suggesting that they can be used safely for local applications.

## 1. Introduction

Throughout history, natural compounds have been used for different types of pathologies including cancer treatment and prevention [1]. Cancer has emerged as a significant health concern in the last few decades, ranking as the first cause of death worldwide [2]. Cancer is defined by the uncontrolled growth of cells, leading to the development of a tumor, either due to alterations in genes encoding cell cycle proteins or as a result of somatic mutations in cell signaling pathways [3]. Due to the fact that cancer cells are able to metastasize and become resistant to radiotherapy and chemotherapy, traditional treatments have become less and less efficient against many malignancies [4]. Even though chemotherapy is the most frequently employed therapeutic approach across various cancer treatments, numerous studies have documented the drawbacks of conventional chemotherapeutic agents, including nonspecific distribution, which results in poor bioavailability, rapid blood clearance, and relatively low solubility in bodily fluids [5]. For half a century, one main focus of the research on natural compounds has been their anticancer potential [6]. Due to their properties to significantly reduce cell resistance against cancer therapies they are frequently used as adjuvants in cancer therapy [7]. The anticancer mechanism of biologically active natural compounds can vary greatly even among the same subclass. One mechanism by which natural compounds like pentacyclic triterpenes exert their cytotoxic effects against cancer cells employs triggering apoptosis in the target cell [8]. The Bcl-2 family of proteins are responsible for the regulation of the intrinsic pathway of apoptosis. Anti-apoptotic proteins like Bcl-2 and Bcl-XL increase cell survival by preventing the release of mitochondrial cytochrome, thereby inhibiting caspase activation. Subsequently, pro-apoptotic proteins promote apoptosis by facilitating cytochrome c release [9]. NF-κB also plays a pivotal role in regulating cell survival through apoptosis. NF-κB increases cell survivability by inducing the upregulation of anti-apoptotic genes, such as inhibitors of apoptosis proteins (IAPs), Bcl-2 protein family members (Bcl-2 and Bcl-XL), and other survival proteins like A20 and GADD45β [10]. Consequently, the inhibition of these targets induces apoptosis, leading to cell death. 

Betulinic acid (BA) is a pentacyclic triterpene naturally occurring in the outer bark of various tree species, occupying a special place among natural remedies [11]. Its pharmacological effects have been extensively researched ever since the discovery of Pisha et al. [12], which proved BA’s selective cytotoxic effects against human melanoma. Not only does BA act as a potent anticancer agent, but it also possesses strong anti-inflammatory, antiviral, antifungal, antidiabetic, renoprotective, cardioprotective, and neuroprotective effects [13,14,15]. One mechanism by which BA exerts its pharmacological effects is through the induction of apoptosis in cancer cells. Some of the pro-apoptotic mechanisms of BA include the inhibition of signaling pathways by downregulating or inhibiting anti-apoptotic players like Bcl-2, Bcl-XL, NF-κB, or cyclin D1 [16,17,18]. Despite its mechanistic complexity, one major drawback is represented by its poor oral bioavailability that hampers its pharmacological potential [19]. In order to overcome this setback, and increase its pharmacologic potential, several strategies have been implemented such as cyclodextrin complexation [20], chemical derivatizations [21], and inclusion in different types of nanoformulations like liposomes, nanoparticles, nanoemulsions, or carbon nanotubes [22]. Chemical modulation includes the possibility to synthesize bioconjugates with various molecules that also exhibit biologic potential; in this regard, fatty acids have been identified as potent antibacterial [23,24], antifungal [25], and anticancer agents [26,27]. Due to the presence of highly reactive carboxylic groups in their structure, which are able to form stable amidic or ester bonds through the conjugation with amino- or hydroxyl-bearing compounds, fatty acids can be employed to generate bioconjugates with significant pharmacological effects [28]. Such chemical derivatizations have been conducted in order to achieve higher biocompatibility with cell membranes [29]. Li et al. have reported the synthesis of a doxorubicin–palmitic acid lipophilic pro-drug by employing a pH-responsive hydrazone linker to conjugate the lipid with doxorubicin and assessed its encapsulation into micelles. The results revealed that doxorubicin conjugation with palmitic acid significantly improved the compatibility and interactions with the lipidic core of the nanocarriers and might facilitate the release of the active compound inside cancer cells [30]. 

Triterpene esterification with fatty acids has thus become an interesting approach in the design of anticancer compounds with improved pharmaco-toxicological profiles. As an example, El-Desouky has isolated five pentacyclic triterpenes, including lupeol tricosanoate from *Salvadora persica* seeds, and tested their cytotoxic activity against MCF-7 breast cancer cells, HT-20 colon cancer cells, and HepG2 hepatocellular carcinoma cells [31]. The in vitro cytotoxic assay revealed that the fatty ester exhibited strong cytotoxic effects against the tested cell lines compared to other pentacyclic triterpenes in free form, displaying IC_50_ values of 9.4 μg/mL (MCF-7 cells), 6.85 μg/mL (HT-29 cells), and 12.74 μg/mL (HepG2 cells).

**Figure 1:**
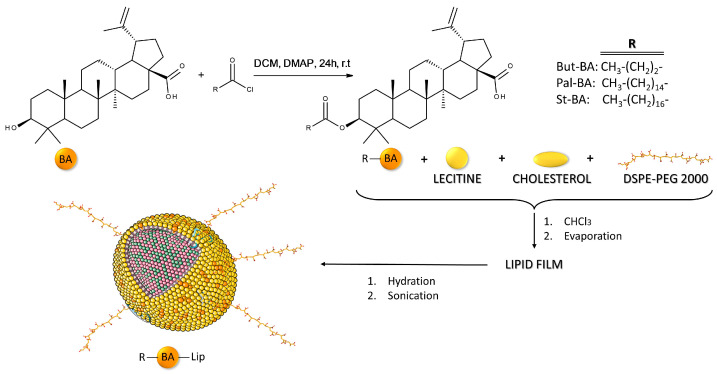
Brief overview of the methodology used for obtaining BA fatty acid esters and their liposomal formulations.

To enhance the anticancer activity of BA, our team has previously synthesized fatty esters of BA with palmitic acid (Pal-BA), stearic acid (St-BA), and butyric acid (But-BA), followed by their inclusion in surface-modified liposomes bearing polyethylene glycol moieties (Pal-BA-Lip, St-BA-Lip, But-BA-Lip) [32]. The methods used for obtaining BA fatty acid esters and their liposomal formulations were previously described in detail [32] and are briefly depicted in Figure 1. The BA fatty esters as well as their liposomal formulations acted as cytotoxic agents against A375 melanoma cells in a dose-dependent manner, while exerting no pro-apoptotic effects in non-malignant human keratinocytes.

Consequently, our current study proposes to further explore their cytotoxic activity against other types of cancer cells, namely breast, colon, and lung cancer cells. Hence, the objectives of our investigation were as follows: (i) to assess the anticancer potential of BA fatty esters against HT-29 human colorectal adenocarcinoma, MCF-7 human breast adenocarcinoma, and NCI-H460 non-small cell lung adenocarcinoma cell lines; (ii) to conduct a docking-based in silico identification of the potential biological targets for both BA and its esters by targeting anti-apoptotic inducers like Bcl-2, Bcl-XL, or NF-κB; and (iii) to conduct a preliminary toxicity assessment through the HET-CAM assay in order to assess the irritative potential of the tested compounds.

## 2. Results

### 2.1. The Evaluation of the Cytotoxic Effect of Betulinic Acid Fatty Esters and Their Liposomes 

**Figure 2:**
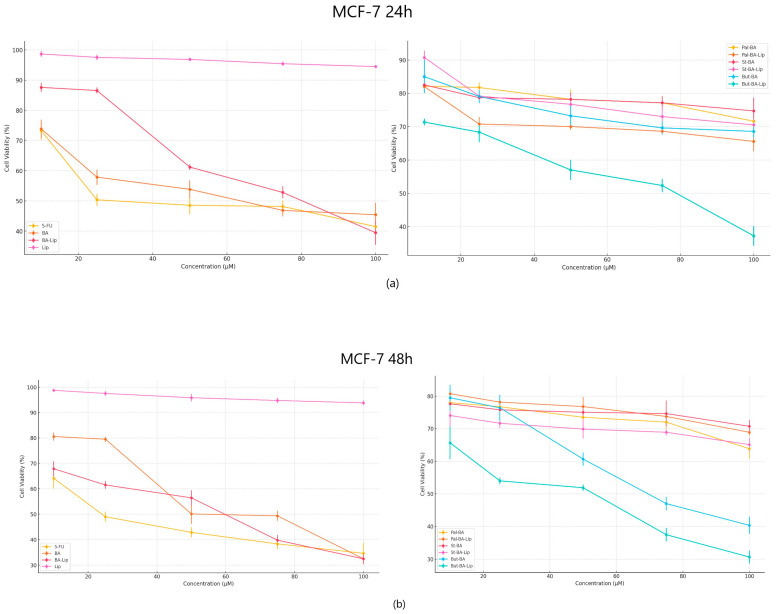
The MCF-7 cell viability ± SD values of three independent experiments 24 h (**a**) and 48 h (**b**) post treatment with BA, But-BA, Pal-BA, St-BA, BA-Lip, But-BA-Lip, Pal-BA-Lip, St-BA-Lip, and 5-FU.

The cytotoxic effect against human colorectal adenocarcinoma HT-29, human breast adenocarcinoma MCF-7, and non-small cell lung adenocarcinoma NCI-H460 cells was evaluated 24 h and 48 h post treatment with the tested compounds (10, 25, 50, 75, and 100 μΜ) by means of the Alamar blue assay. The incubation of human breast adenocarcinoma MCF-7 cells for 24 and 48 h revealed that But-BA-Lip significantly decreased cell viability, with reductions to 37.23% after 24 h and 30.76% cell viability percentage after 48 h compared to the control (100%) and compared to the parent compound, BA and its liposome, BA-Lip, displaying lower IC_50_ values after 48 h (44.88 μM for But-BA-Lip, 54.97 μM for BA, and 54.89 μM for BA-Lip) (Table 1). However, none of the tested compounds was able to match the antiproliferative activity of 5-fluorouracil (5-FU) (30.79 μM) (Figure 2a,b). Moreover, it was revealed that all liposomes exerted stronger cytotoxic effects compared to their respective fatty esters when the highest concentrations were applied (Table 2).

**Figure 3:**
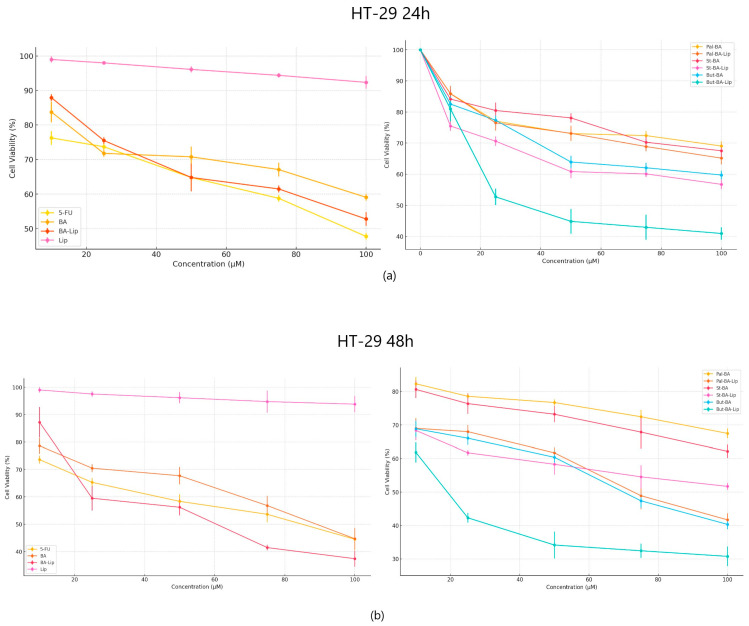
The HT-29 cell viability ± SD values of three independent experiments 24 h (**a**) and 48 h (**b**) post treatment with BA, But-BA, Pal-BA, St-BA, BA-Lip, But-BA-Lip, Pal-BA-Lip, St-BA-Lip, and 5-FU.

In terms of their anticancer activity against colon cancer, all liposomes, except St-BA-Lip, exhibited more potent antiproliferative effects than their respective free esters (70.06 μM for Pal-BA-Lip, 30.57 μM for But-BA-Lip, and 59.04 μM for BA-Lip); they were also able to inhibit cancer cell growth more aggressively than BA (91.16 μM) and 5-FU (82.53 μM) (Figure 3a,b). The liposomes induced decreased cell viability (%) at an accelerated rate at the highest concentrations, well superior to the free esters, BA, and 5-FU, 24 h and 48 h post treatment (Table 2).

**Figure 4:**
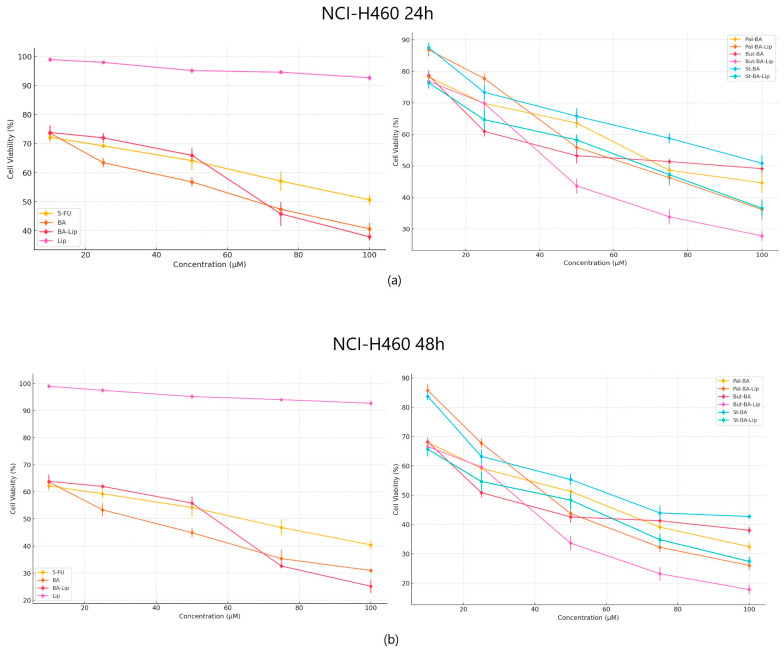
The NCI-H460 cell viability ± SD values of three independent experiments 24 h (**a**) and 48 h (**b**) post treatment with BA, But-BA, Pal-BA, St-BA, BA-Lip, But-BA-Lip, Pal-BA-Lip, St-BA-Lip, and 5-FU.

When tested against NCI-H460 non-small cell lung cancer, both the esters and their liposomes exhibited stronger cytotoxic effects compared to 5-FU (Figure 4a,b) used as positive control; furthermore, while comparing the antiproliferative effects against NCI-H460 cells, it was revealed that all liposomes acted as stronger antiproliferative agents compared to their free esters (Table 2). Free liposomes did not exhibit cytotoxic effects against either tested cancer cell line.

### 2.2. Fatty Ester Derivatives on Cell Morphology 

**Figure 5:**
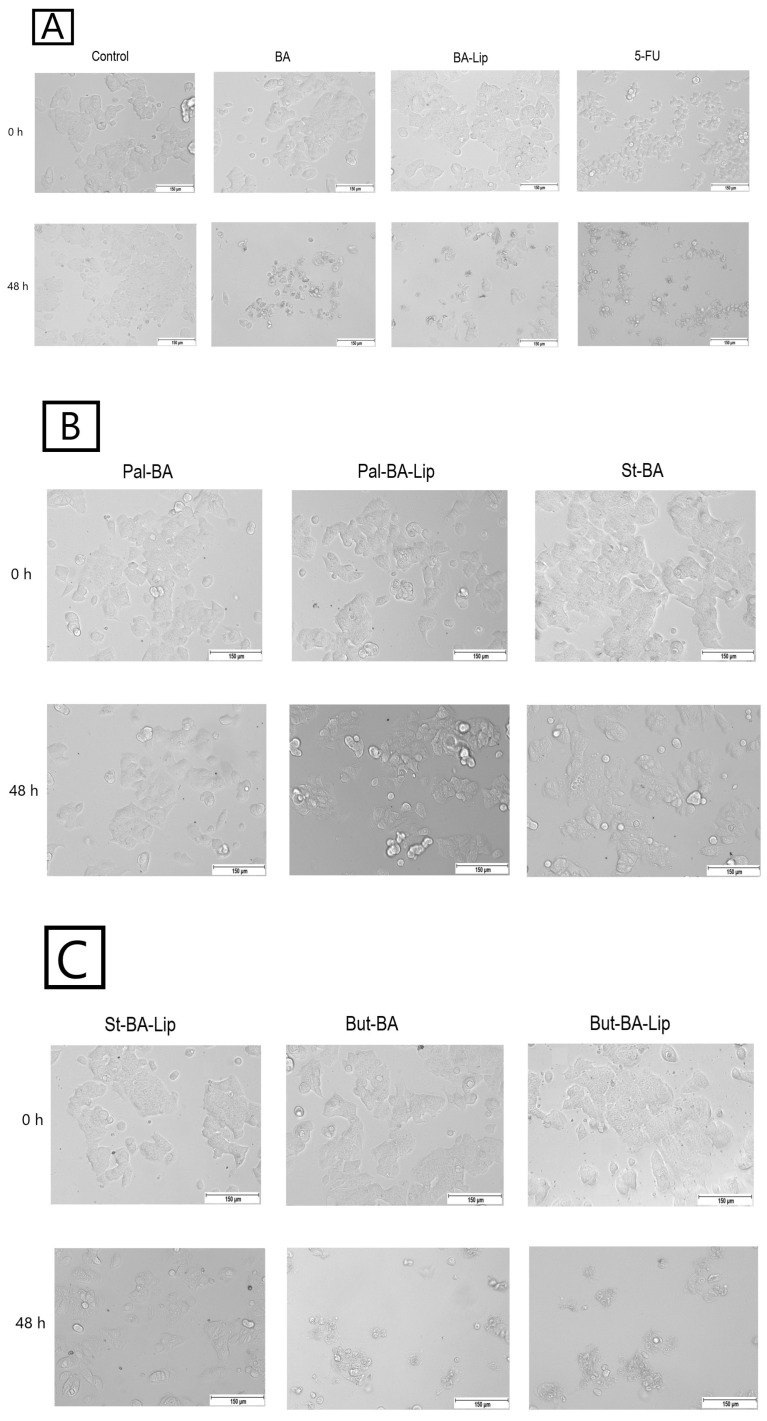
The effects of BA, BA-Lip, and 5-FU (IC_50_) (**A**), Pal-BA, Pal-BA-Lip, and St-BA (IC_50_) (**B**), and St-BA-Lip, But-BA, and But-BA-Lip (IC_50_) (**C**) on human breast adenocarcinoma MCF-7 cells’ morphology both 0 h and 48 h after stimulation; the scale bar was 150 μm.

**Figure 6:**
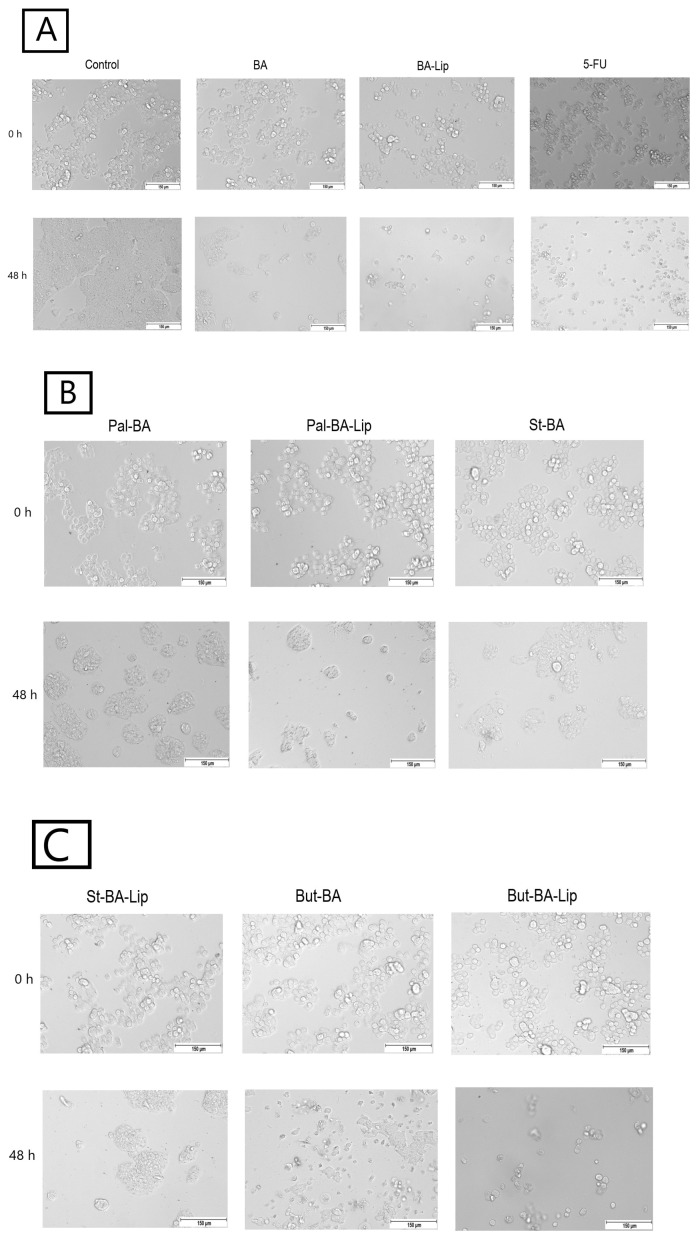
The effects of BA, BA-Lip, and 5-FU (IC_50_) (**A**), Pal-BA, Pal-BA-Lip, and St-BA (IC_50_) (**B**), and St-BA-Lip, But-BA, and But-BA-Lip (IC_50_) (**C**) on human colorectal adenocarcinoma HT-29 cells’ morphology both 0 h and 48 h after stimulation; the scale bar was 150 μm.

**Figure 7:**
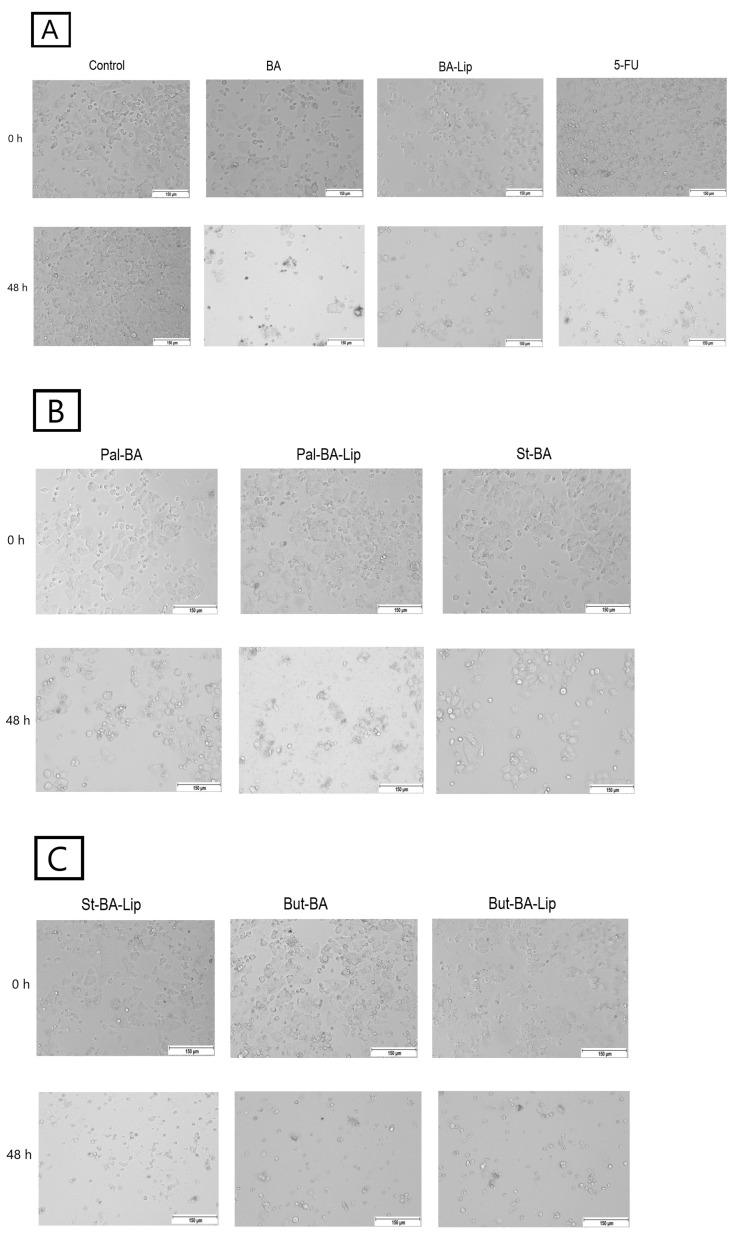
The effects of BA, BA-Lip, and 5-FU (IC_50_) (**A**), Pal-BA, Pal-BA-Lip, and St-BA (IC_50_) (**B**), and St-BA-Lip, But-BA, and But-BA-Lip (IC_50_) (**C**) on non-small cell lung adenocarcinoma NCI-H460 cells’ morphology both 0 h and 48 h after stimulation; the scale bar was 150 μm.

In MCF-7, HT-29, and NCI-H460 cells, treatment with the concentrations corresponding to the IC_50_ values of each ester (Pal-BA, St-BA, and But-BA) and their respective liposomes (Pal-BA-Lip, St-BA-Lip, and But-BA-Lip) induced morphological changes in accordance to the recorded viability results, showing a significant number of round and detached cells, correlated with a lower number of cells compared to the control group (Figure 5, Figure 6 and Figure 7).

To assess the cytotoxic effects recorded in MCF-7, HT-29, and NCI-H460 cells after the 48 h of treatment with the fatty esters as well as with their liposomal formulations with the corresponding IC_50_ values, the cells’ nuclei were stained with Hoechst solution, while the cytoskeletons were labeled with F-actin. Furthermore, the CellEvent caspase-3/-7 green detection staining was employed in order to analyze whether the caspase activity increased post treatment. 

**Figure 8:**
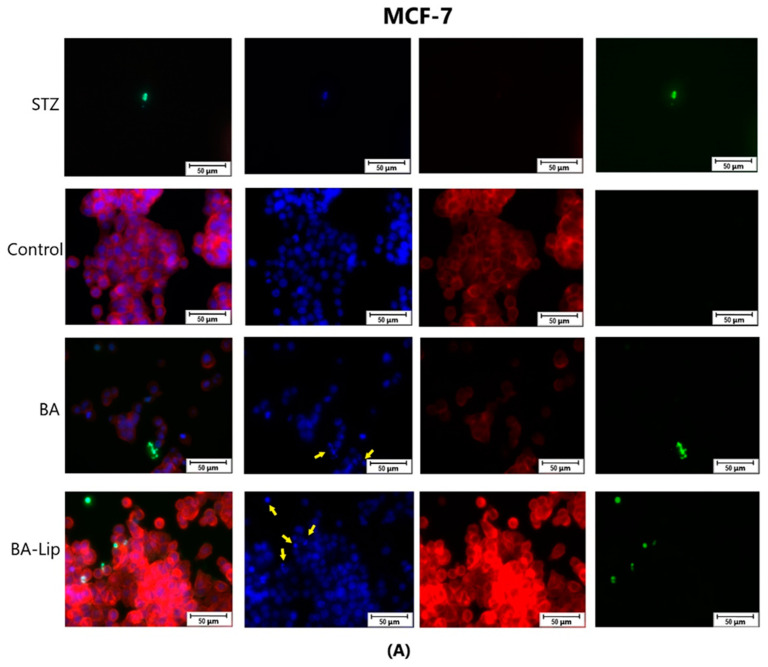

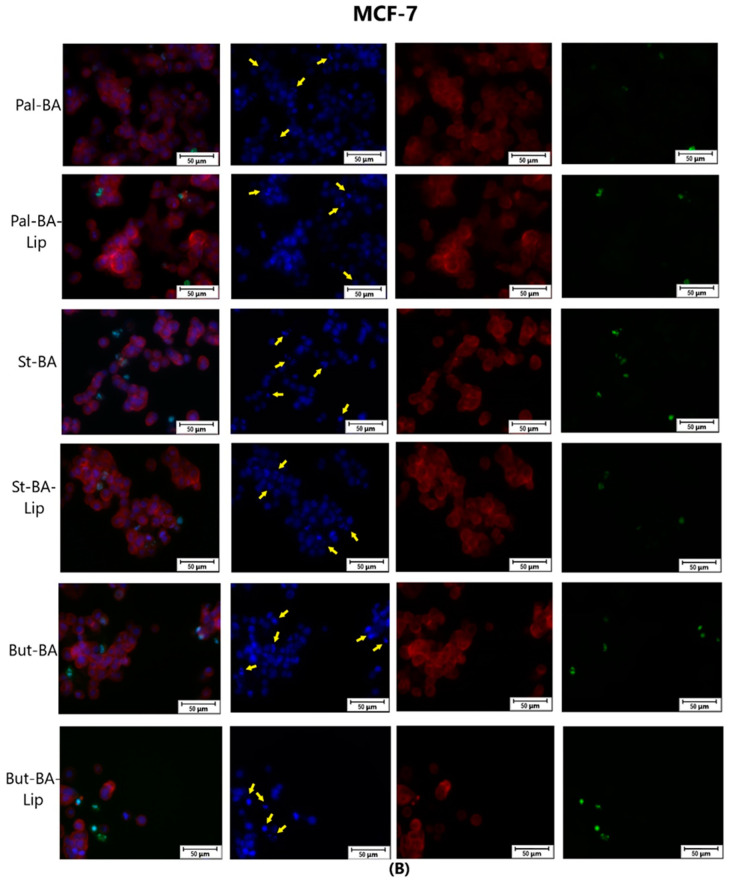
The effect of BA-Lip and BA (**A**) (IC_50_ values) and of Pal-BA, St-BA, But-BA, Pal-BA-Lip, St-BA-Lip, and But-BA-Lip (**B**) (IC_50_) on nuclei (Hoechst—blue staining), cytoskeleton (F-actin—red staining), and caspase-3/-7 signals (CellEvent—green staining) on MCF-7 cells. For comparing apoptosis with necrotic death induction, the stimulation with staurosporine (STZ 1 μM) was used. The yellow arrows in the second column indicate signs of apoptotic cell death, while the bright green spots from the fourth column indicate an increased caspase-3/-7 activity. The scale bar was 50 μm.

**Figure 9:**
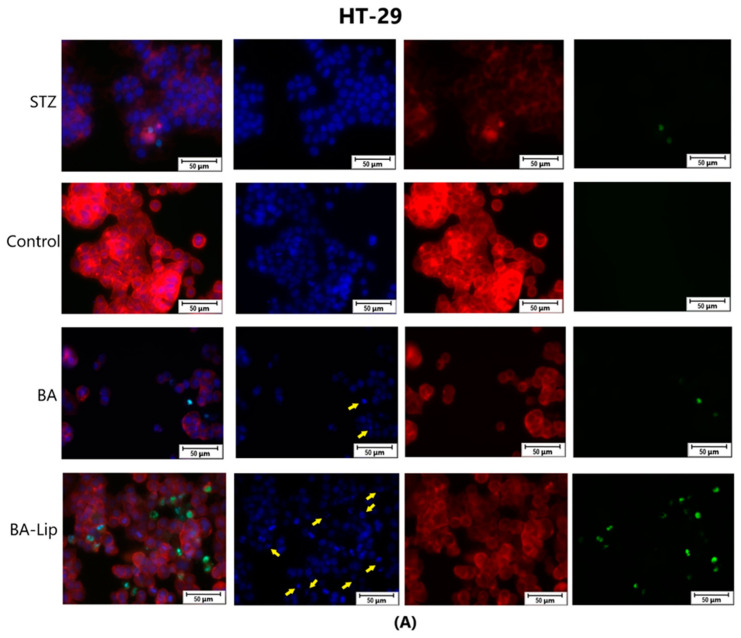

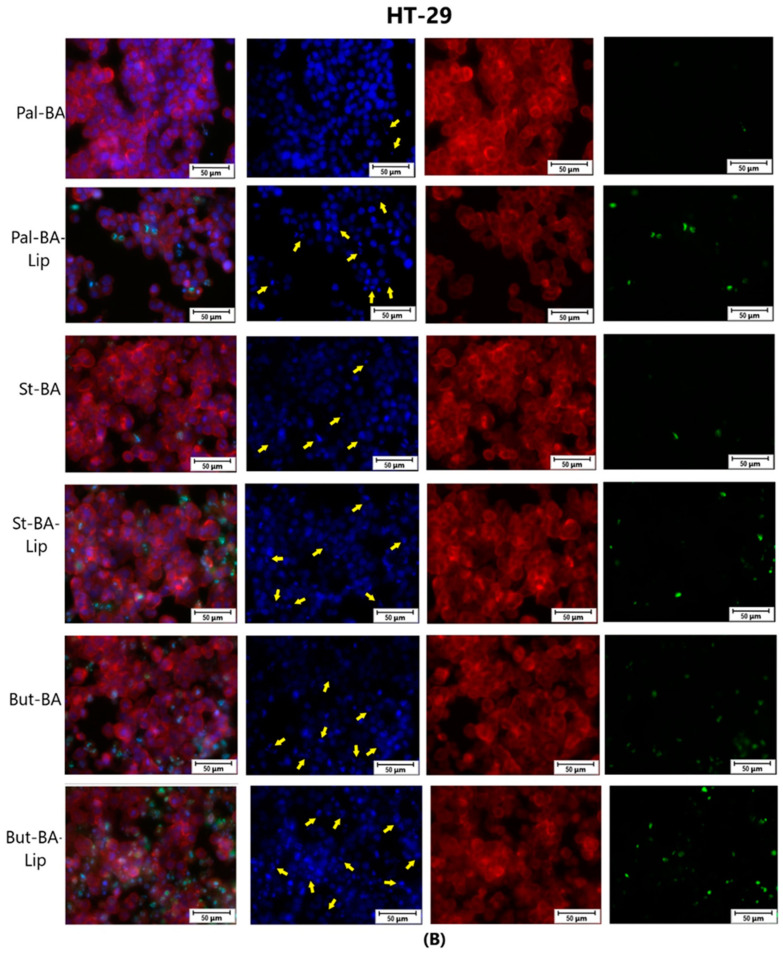
The effect of BA-Lip and BA (**A**) (IC_50_ values) and of Pal-BA, St-BA, But-BA, Pal-BA-Lip, St-BA-Lip, and But-BA-Lip (**B**) (IC_50_) on nuclei (Hoechst—blue staining), cytoskeleton (F-actin—red staining), and caspase-3/-7 signals (CellEvent—green staining) on HT-29 cells. For comparing apoptosis with necrotic death induction, the stimulation with staurosporine (STZ 1 μM) was used. The yellow arrows in the second column indicate signs of apoptotic cell death, while the bright green spots from the fourth column indicate an increased caspase-3/-7 activity. The scale bar was 50 μm.

**Figure 10:**
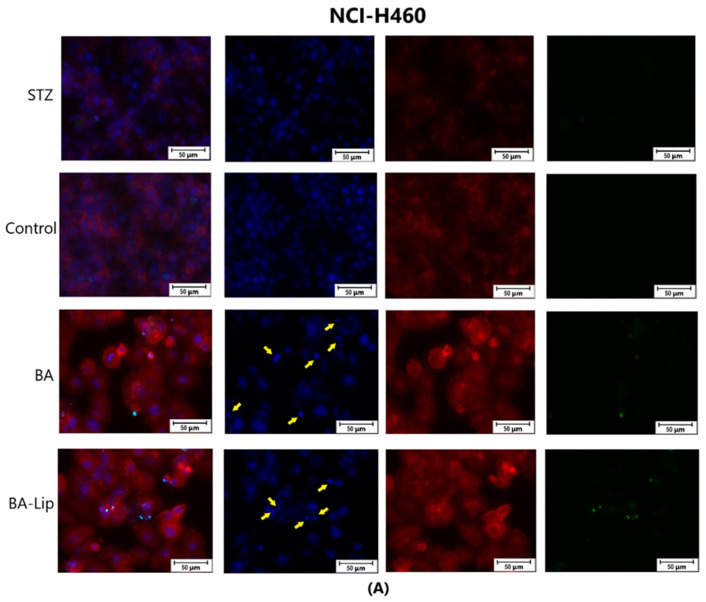

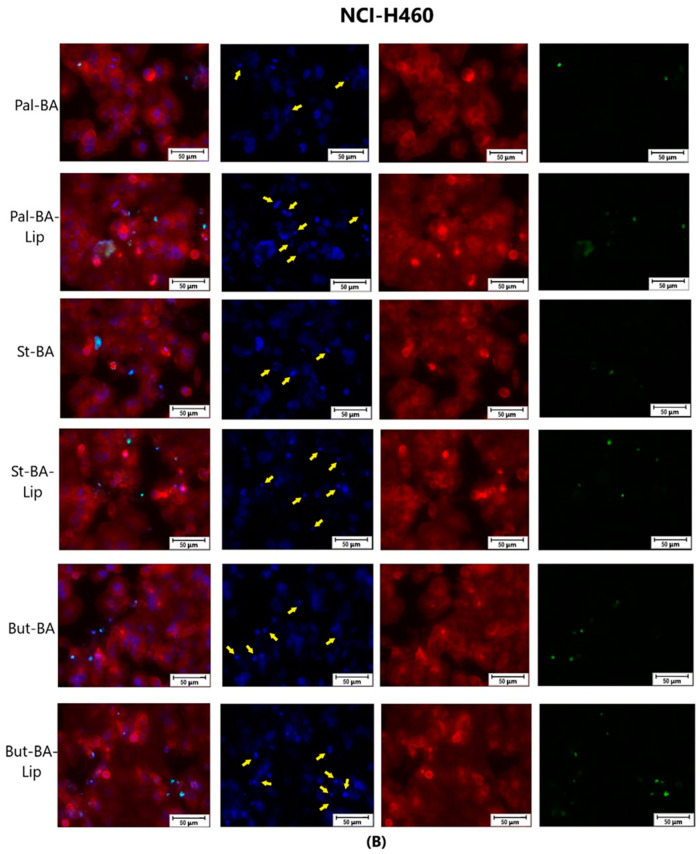
The effect of BA-Lip and BA (**A**) (IC_50_ values) and of Pal-BA, St-BA, But-BA, Pal-BA-Lip, St-BA-Lip, and But-BA-Lip (**B**) (IC_50_) on nuclei (Hoechst—blue staining), cytoskeleton (F-actin—red staining), and caspase-3/-7 signals (CellEvent—green staining) on NCI-H460 cells. For comparing apoptosis with necrotic death induction, the stimulation with staurosporine (STZ 1 μM) was used. The yellow arrows, in the second column indicate signs of apoptotic cell death, while the bright green spots from the fourth column indicate an increased caspase-3/-7 activity. The scale bar was 50 μm.

Specific apoptotic hallmarks were observed in all cells treated with both the esters and the liposomes, shown as small and bright nuclei, small and round-shaped cells, and nuclear fragmentations, after being stained with the Hoechst solution. By employing the CellEvent caspase-3/-7, it was revealed that the activated caspase-3/-7 signals, appearing as bright green spots overlapping the bright nuclei of the dead cells (Hoechst), were significantly increased after 48 h of treatment with the tested compounds (Figure 8, Figure 9 and Figure 10).

### 2.3. HET-CAM Assay 

**Figure 11:**
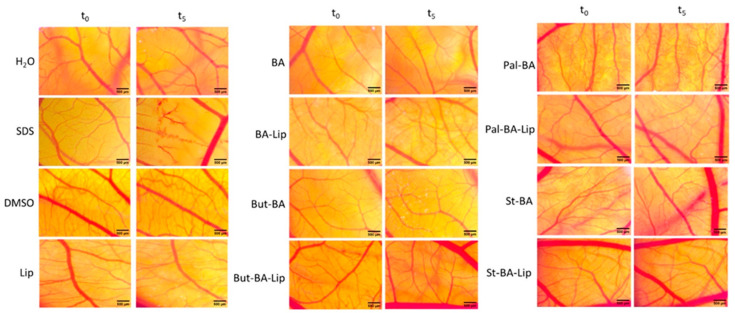
The HET-CAM method-based irritation test. Stereomicroscope images of the chorioallantoic membrane were captured before (T0) and 300 s (T5) post treatment with 600 μL BA, BA-Lip, But-BA, But-BA-Lip, Pal-BA, Pal-BA-Lip, St-BA, St-BA-Lip, and Lip (tested at 100 μM); distilled water and sodium dodecylsulfate (SDS) 0.5%, were used as negative and positive control, respectively. Scale bars were set at 500 μm.

The HET-CAM test was used to assess the irritative potential of BA’s fatty esters and their liposomal formulations (Figure 11).

The irritation potential of each compound was investigated using the Luepke scale [33] which assigns the tested compounds values between 0 and 21 due to the reaction of the chorioallantoic membrane. Non-irritant compounds exhibit values between 0 and 0.9; slightly irritant compounds between 1 and 4.9; moderate irritative compounds are indicated by scores of 5 to 8.9, and strongly irritant compounds have scores between 9 and 21. According to our findings (Table 3), neither the fatty esters (Pal-BA, St-BA, But-BA), nor the liposomal formulations (Pal-BA-Lip, St-BA-Lip, But-BA-Lip) exhibited any sign of irritation. There was no occurrence of hemorrhage, coagulation, or vessel disruption during the 5 min observation time. Moreover, all samples showed good tolerability even 24 h post treatment, leading to the conclusion that they would be appropriate for cutaneous and mucosal applications. 

### 2.4. Molecular Docking 

Molecular docking is a useful tool that can help you understand the potential protein-targeted molecular mechanism of action of certain active biological compounds. BA and similar derivatives have previously been shown to target anti-apoptotic proteins such as Bcl-2, Bcl-XL, or NF-κB exerting their pro-apoptotic effect through this mechanism [17,18,34]. Previous reports suggest that certain chemical modulations of various triterpenic scaffolds increase their theoretical affinity for targets such as Bcl-2, Bcl-XL, and NF-κB [35,36,37,38]. As a result, we used a two-way molecular docking approach, employing two different scoring functions (Vina and Glide), to determine whether fatty acid esters of BA are more prone to theoretically inhibit these anti-apoptotic proteins. Obtained docking scores are available in Table 4. Glide generates multiple docking scores after a docking instance is finished. In this case, despite the fact that the developers claim that the Emodel score is considerably better at identifying a suitable pose than either the molecular mechanics energy or GlideScore alone [39], we used GlideScore because it gave the same ranking and nearly identical docking poses.

**Figure 12:**
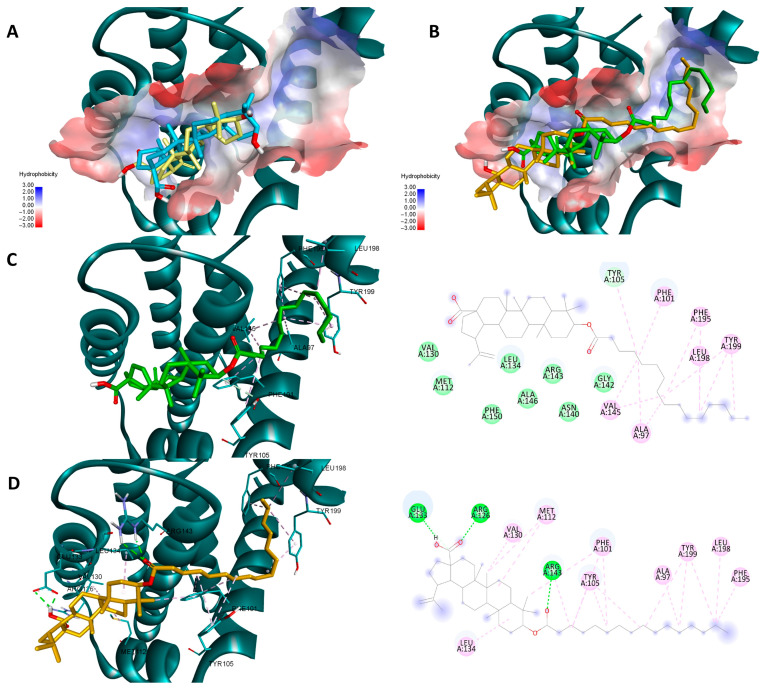
Overlaid docking poses of BA, generated by Vina (yellow) and Glide (blue), within the binding site of Bcl-2 (**A**); overlaid docking poses of Pal-BA, generated by Vina (green) and Glide (orange), within the binding site of Bcl-2 (**B**); 3D and 2D depictions of Pal-BA–Bcl-2 interactions of the Glide-generated pose (**C**) and Vina-generated pose (**D**); hydrogen bonds are depicted as green dotted lines while hydrophobic interactions are depicted as pink dotted lines; all images are generated using the 3D structure of Bcl-2 (PDB entry 4LVT).

Both sets of docking results obtained for NF-κB show opposing rankings of the four docked compounds. Glide scores the long-side-chain-containing compounds (Pal-BA, St-BA) better whereas Vina ranks the smaller compounds (BA, But-BA) higher. These differences may arise due to several factors related to the size of the Rel-Homology-Domain (RHD) within NF-κB p50 used for docking, and the different scoring functions used by both docking programs. Glide’s scoring function favors hydrophobic interaction and shape complementarity [39]. Given their large hydrophobic flexible side chain, Pal-Ba and St-BA may benefit when docked with glide. Vina’s scoring function balances hydrogen, hydrophobic, and electrostatic binding differently and thus can favor the smaller compounds due to their better complementarity with available sub-pockets, achieved through other types of interactions, not just hydrophobic ones [40]. Nevertheless, for this case we can conclude that this type of chemical derivatization does not improve BA’s significant binding towards NF-κB, nor is this protein a druggable target for these types of compounds. In the case of the other two targets, results show that for both proteins and docking scores, longer fatty acid esters are preferred over BA or But-BA. Overall, based on all four cases, Pal-BA emerged as the best potential inhibitor for both proteins. Slight ranking order variations may again arise due to the difference between the two scoring functions used by both programs. Nevertheless, Pal-BA or St-BA being more theoretically active then BA or But-BA was expected in this case, because the binding sites of Bcl-2 and Bcl-XL are large mostly hydrophobic pockets, consisting of four binding grooves named p1, p2, p2, and p4 [41]. This is evident in the case of Bcl-XL, where Vina and Glide positioned BA roughly in the same region of the binding site. However, due to its compact structure, it is unable to bind to the p4 pocket, which is visible in the upper right region of the binding site [42] (Figure 12A). Both generated poses of Pal-BA (by Vina and Glide), have the extended hydrophobic fatty acid side chain available to occupy the p4 pocket, giving it an extra affinity edge over BA (Figure 12B). Similarly, the presence of the fatty acid side chain causes several hydrophobic interactions within the p4 binding spot, for both generated poses of Pal-BA (Figure 12C,D). There is one distinction worth noting. While the Vina pose does not interact via hydrogen bonds (HB), the Glide pose does, using both oxygen-containing sites to form HBs with ARG126, GLU 133, and ARG 143 (Figure 12C,D).

**Figure 13:**
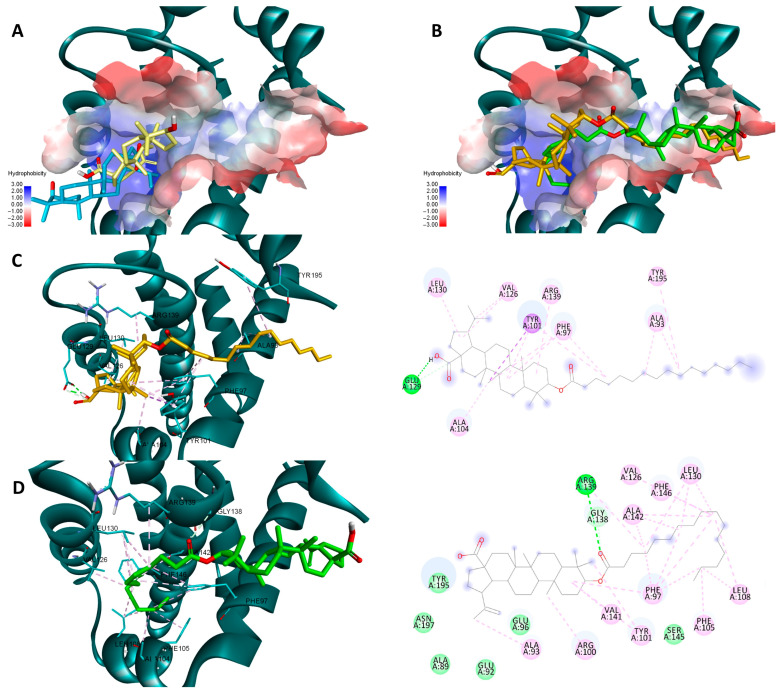
Overlaid docking poses of BA, generated by Vina (yellow) and Glide (blue), within the binding site of Bcl-XL (**A**); overlaid docking poses of Pal-BA, generated by Vina (green) and Glide (orange), within the binding site of Bcl-XL (**B**); 3D and 2D depictions of Pal-BA-Bcl-XL interactions of the Glide-generated pose (**C**) and Vina-generated pose (**D**); hydrogen bonds are depicted as green dotted lines while hydrophobic interactions are depicted as purple/pink dotted lines; all images are generated using the 3D structure of Bcl-XL (PDB entry 2YXJ).

Pal-BA outperforms BA in terms of theoretical affinity in the case Bcl-XL for the same reason. BA, being docked at the p2 site, leaves the p4 pocket empty (Figure 13A). Pal-BA has a larger structure than BA, allowing it to occupy both the p2 and p4 pockets (Figure 13B), which are required for Bcl-Xl inhibition [43]. There are some differences between Pal-BA’s two generated poses. The Vina-generated pose depicts Pal-BA’s fatty acid side chain entering the p2 pocket and the triterpene end binding in the p4 region, showing that it favors for hydrophobic interaction formation, while the esters’ C=O form a HB with ARG 139. The Glide pose is reversed, with the acid tail bound at the p4 site and the triterpene end on p2. However, because a HB between COOH and GLU129 was preferred, the hydrophobic isopropenyl group did not bind as deeply into the p2 pocket as the hydrophobic acid residue did in the Vina-generated pose (Figure 13C,D).

## 3. Discussion

Betulinic acid is a pentacyclic triterpene widely distributed in the plant kingdom, mostly found in the outer bark of numerous tree species, exhibiting a plethora of biological activities [44]. BA was reported initially as a selective cytotoxic agent against human melanoma [12]; subsequent studies demonstrated its anticancer effects against a wide range of human cancers such as neuroectodermal tumors (glioblastoma, medulloblastoma, neuroblastoma), breast, colon, hepatocellular, lung, prostate, ovarian, renal, and head and neck carcinomas [45,46,47]. Literature has reported that fatty acid analogues are associated with a wide range of pharmacological effects such as anti-inflammatory, antioxidant, and neuroprotective [48,49] and have even shown promising potential in cancer treatment [50]. It is a well-recognized strategy that creating new molecular hybrids through the conjugation of two active biological agents might result in highly improved pharmacological effects. Therefore, taking into account the biological effects of both BA and fatty acids, the combination of these molecules through ester bonds might lead to significantly enhanced anticancer activities [26]. In a similar manner, Pinzaru et al. have synthesized long-chain fatty acids esters of betulin and BA using myristoyl chloride as reaction partner and tested them against A431 skin epidermoid carcinoma cells and A375 human melanoma cells; the results showed that both fatty esters of BA and betulin exhibited stronger cytotoxic effects against both tested cell lines compared to the parent compounds [51]. 

In order to achieve enhanced bioavailability, BA’s fatty esters were incorporated into liposomes with a modified surface using polyethylene glycol (PEG) moieties. Liu et al. have assessed PEGylated BA liposomes in vitro against HepG2 and HeLa cells where they exhibited superior effects against both HepG2 cells (inhibitory rate of 86.98% after 72 h) and HeLa cells (88.89% after 72 h) compared to free BA or BA conventional liposomes; when tested in vivo in U14 tumor-bearing mice the PEGylated BA liposomes were the most efficient in inhibiting tumor growth, compared to bare BA and BA incorporated in conventional liposomes, while not causing any toxicity to the experimental animals [52]. Our team has previously tested the cytotoxic effects of St-BA, Pal-BA, and But-BA, and their liposomes, St-BA-Lip, Pal-BA-Lip, and But-BA-Lip, against A375 and HaCaT cells [32]. The results revealed that both the esters and their liposomal formulations acted as potent A375 cytotoxicity inducers after prolonged exposure with the highest concentrations, while St-BA-Lip, But-BA, and But-BA-Lip displayed lower IC_50_ values compared to the free BA. Results also showed that, except for the highest tested concentration of But-BA and But-BA-Lip, none of the synthesized compounds exhibited cytotoxic effects against HaCaT cells regardless of the used concentration. Consequently, we have further assessed their anticancer potential in vitro against breast, colon, and lung cancer cells lines. By means of the Alamar Blue assay, the BA fatty acids’ esters (Pal-BA, St-BA, But-BA) as well as their liposomal formulations (Pal-BA-Lip, St-BA-Lip, But-BA-Lip) were tested against human breast adenocarcinoma MCF-7, human colorectal adenocarcinoma HT-29, and non-small cell lung adenocarcinoma NCI-H460 cells in terms of cell viability; all the compounds displayed cytotoxic activities in a time- and dose-dependent manner. 

The cytotoxicity assessment against MCF-7, HT-29, and NCI-H460 cells revealed that all the liposomes exerted better cytotoxic effects compared to their corresponding BA fatty acid esters. It was also shown that But-BA-Lip outperformed the parent compound (BA) and its liposomal formulation, BA-Lip, against all tested cancer cells; moreover, against NCI-H460 and HT-29 cells But-BA-Lip was able to exhibit much improved IC_50_ values compared to the positive control 5-FU. 

Our results are consistent with the ones found in literature where BA has been assessed against MCF-7 cells (IC_50_ 112 μM 48 h post treatment) [53], HT-29 cells (IC_50_ 84.5 μM 48 h after treatment) [54], and NCI-H460 cells (EC_50_ 6.1 μg/mL 48 h post treatment) [55]. Furthermore, BA’s inclusion in liposomes induced stronger in vitro anticancer effects against HepG2 liver cancer cells compared to free BA [52]. Calculated IC_50_ values for BA liposomes and BA liposomes modified with biosurfactant MEL-A were 6.02 g/mL and 3.11 g/mL, respectively; the formulations were tested against HepG2 cells, thereby revealing a significantly increased anticancer activity as a result of liposome encapsulation [56]. 

Over the last few years, a plethora of studies aimed to elucidate the molecular mechanisms underlying the BA-mediated anticancer activity. One common feature of BA cytotoxic effects is its ability to modulate the mitochondrial apoptotic pathway in cancer cells [57]. Cell death can occur through various distinct processes; apoptosis is one such process, which can be caused by both intrinsic, through mitochondrial pathways, and extrinsic events, through interactions with extracellular factors [58]. Yaouzu et al. have explored the molecular mechanisms of BA antiproliferative effects against U87MG and A172 glioblastoma cells revealing the downregulation of the NF-κB pathway and upregulation of caspase-3 and -9, thus suggesting that apoptosis occurred through mitochondria-mediated mechanisms [59]. Additionally, several complementary mechanisms were identified such as the modulation of caspase-3 and -7 activity, which triggered apoptosis in HepG2 and SMMC-7721 hepatocarcinoma cells [60], A549 and NCI-H1703 non-small cell lung cancer cells [61], and u937 leukemia cells [62]. To establish if BA fatty acid esters, as well as their liposomal formulations, act as pro-apoptotic agents through similar molecular mechanisms as free BA, the concentrations for the corresponding IC_50_ values were selected for each compound to be submitted to caspase-3/-7 as well as Hoechst (nuclei) and F-actin (cytoskeleton) staining. It was found that in all three tested cancer cell lines, both the esters and their liposomal formulations induced morphological changes that are consistent with apoptosis, such as membrane blebbing, nuclear fragmentation, and chromatin condensation, as well as cell shrinkage. After 48 h treatment with the tested compounds, the activated caspase-3/-7 signals were significantly increased compared to the control cells. Palmitate and palmitate derivatives were found to trigger apoptosis via specific effects on mitochondria [63], some studies suggesting that its molecular mechanism relies on the activation of caspase-3 [64]. Butyric acid is able to induce growth arrest and apoptosis in different types of cancer cells [65]. Chopin et al. have revealed that sodium butyrate inhibited proliferation in breast cancer cells by activating Fas-induced apoptosis in a time- and dose-dependent manner; the authors have also emphasized that Fas-induced apoptosis is often enhanced by cytochrome C release and complete processing of caspase-7 [66]. Wang et al. also proved butyric acid’s cytotoxic effects against colorectal adenocarcinoma HT-29 cells [67]; the results indicated that sodium butyrate was able to upregulate the activities of caspase-3 and -9, suggesting that the molecular mechanism of apoptosis induction resides in mitochondrial pathways that do not involve TNF-α. Furthermore, butyric acid inclusion in liposomal formulations has shown strongly improved cytotoxic effects compared to the free compound [68]. Stearate was also found to induce selective apoptosis in breast cancer cells versus non-cancerous cells by inhibiting the activity of protein kinase C [69]. Considering that both BA and the selected fatty esters exert strong pro-apoptotic effects by modulating the caspase’s activity, we may conclude that their association leads to compounds with much improved anticancer activity, while their inclusion in liposomal formulations might facilitate their delivery inside cancer cells. 

The application of the HET-CAM assay represents a reliable way of testing the irritant character of a newly synthesized compound by assessing inflammatory reactions to the CAM, such as lysis, coagulation, and hemorrhage [70]. According to the Luepke score, none of the tested compound showed any irritation potential, hence being suitable for cutaneous and mucosal usage. It was previously revealed that BA had no vascular effect on the CAM model, being classified as a non-irritant compound [71]. Furthermore, Ghiulai et al. have demonstrated that BA conjugation with gold nanoparticles had no adverse effects on any of the three parameters of the vascular plexus of the CAM, making it compatible for local applications [72]. 

Molecular docking was previously used to determine whether the theoretical affinity of BA and other triterpenic acids increases as larger, mostly hydrophobic, groups are attached to the parent compound. A previous study found that condensing the COOH groups of BA, OA, and UA with 1-hydroxibenzotriazole increased the theoretical binding affinity to Bcl-2 [35]. This allowed for a better occupation of Bcl-2’s p2 and p4 hydrophobic pockets. Double-substituted triterpenic structures with large hydrophobic groups on both ends can perform even better. This was the case with a 2,30-bispyridinylidene derivative of 3,20-dioxo-lup-28-oate, which had a higher binding affinity than the co-crystallized Bcl-Xl ligand, ABT-737 [37]. Both pyridinyl groups allowed Bcl-XL’s p2 and p4 binding sites to be occupied efficiently at the same time. For the same reasons as stated above, esterification with higher fatty acids increased BA’s theoretical binding affinity to Bcl-2 and Bcl-XL. However, these data do not correspond to our biological results. There are some possible explanations for why this is the case but for the most part, BA has a complex anticancer mechanism of action. Targeting these two proteins is not the only way BA exerts its pro-apoptotic effects. Other methods include targeting mRNA translation and changing the normal pro- and anti-apoptotic transcript protein ratio [37]. Nevertheless, we may conclude that esterification of BA using large fatty acids may be a way of increasing its binding potential towards anti-apoptotic proteins like Bcl-2 and Bcl-XL. 

Collectively, the biological data reported significant cytotoxic activity for all BA fatty acids esters, with the butyric derivative exerting the greatest results against breast, colon, and lung cancer cell lines. The inclusion of these hybrids in surface-modified liposomal formulations led to lower IC_50_ values and, hence, to increased cytotoxic effects compared to the parent compound (BA).

## 4. Materials and Methods

### 4.1. Cell Culture 

Human colorectal adenocarcinoma HT-29, human breast adenocarcinoma MCF-7, and non-small cell lung adenocarcinoma NCI-H460 cell lines (acquired from American Type Culture Collection ATCC, Lomianki, Poland) were selected for our study. The cells were obtained as frozen items and were stored in liquid nitrogen until further usage. The MCF-7 cells were propagated in Eagle’s Minimum Essential Medium (EMEM) supplemented with 10% fetal bovine serum (FBS), 1% penicillin/streptomycin mixture (100 IU/mL) and 0.01 mg/mL human recombinant insulin. McCoy’s 5A Medium supplemented with 10% FBS and 1% antibiotic mixture was used for HT-29 cell line propagation, while NCI-H460 cells were propagated in RPMI-1640 Medium containing the same concentrations of antibiotic mixture and fetal bovine serum. All cells were incubated under 5% CO_2_ and at 37 °C. 

### 4.2. The Evaluation of Cell Viability

HT-29, NCI-H460, and MCF-7 cell viability was evaluated by employing the Alamar blue colorimetric assay. The cells were seeded at an initial concentration of 10^4^ cells/well into 96-well plates and stimulated for 24 h and 48 h with BA, St-BA, Pal-BA, But-BA, St-BA-Lip, Pal-BA-Lip, But-BA-Lip, and BA-Lip using increasing concentrations, including the concentrations corresponding to BA’s IC_50_ values found in related studies (10, 25, 50, 75, and 100 μM) prepared from 20 mM stock solutions. Firstly, the corresponding quantity of each fatty ester was dissolved in 500 μL DMSO for obtaining a stock solution of 20 mM. Afterwards, the stock was diluted into the specific medium for each cell line to obtain the tested concentrations (10, 25, 50, 75, and 100 μM). The 20 mM stock solutions of each liposomal formulation were prepared in PBS and then diluted in the specific medium for each cell line for obtaining the desired concentrations. Some wells were treated with free liposome (Lip) and with 5-fluorouracil (5-FU) as positive anticancer agent. The DMSO final concentration did not surpass 0.5%. An automated cell counting device was utilized along with Trypan blue coloring for determining the cell number (Thermo Fisher Scientific, Inc., Waltham, MA, USA). Alamar blue 0.01% coloring was employed after 24 h and 48 h for staining all cells, which were afterwards incubated for another 3 h. An xMarkTM Microplate Spectrophotometer Bio-Rad (Hercules, CA, USA) was utilized for determining the absorbance measurements, employing 2 wavelengths (570 nm, 600 nm). All experiments were performed in triplicates on separate plates and finally the average values were determined from three individual experiments. The viabilities were calculated using specific statistical tests, by employing the GraphPad Prism version 6.0.0 software, by comparing the obtained absorbance values of the treated cells with the control group. 

### 4.3. Cell Morphology

The MCF-7, HT-29, and NCI-H460 cells were seeded into 12-well plates at an initial density of 10^5^ cells/well. After reaching 80–85% confluence, the old medium was removed and the cells were stimulated with BA’s fatty esters and their respective liposomal formulations for 48 h using their corresponding IC_50_ values for each cell line. All the cells were stimulated with 5-FU as positive control. The cell morphology was assessed using the EVOS™ M5000 Imaging System equipped with a highly sensitive CMOS camera (Thermo Fisher Scientific, Inc., Waltham, MA, USA).

### 4.4. Immunofluorescence Assay—Morphological Assessment of Apoptotic Cells

The evaluation of nuclear localization and any signs of apoptosis (shrinkage, fragmentation) were determined using Hoechst staining and caspase-3/-7 cell event, while the cytoplasmic localization and alterations were determined using F-actin staining. MCF-7, HT-29, and NCI-H460 cells were seeded into 12-well plates at 2 × 10^5^ cells/well initial density and cultured until reaching 80–85% confluence. Afterwards, the old media was removed and the cells were stimulated with the IC_50_ values corresponding to each compound for 48 h. Separately, some wells were stimulated with staurosporine 1 μM as positive control. After 48 h, the old medium was removed using an aspiration station and replaced with caspase-3/-7 Green detection agent, 5 μM in complete medium specific to each cell line, and the cells were incubated for 30 min at 37 °C under a 5% CO_2_ atmosphere. Afterwards, the medium with caspase-3/-7 Green detection agent was removed using an aspiration station and the cells were fixed with paraformaldehyde, 3%, for 15 min and permeabilized with Triton X, 0.1% in PBS, for 5 min. Subsequently, the cells were stained with 100 μL 1 X Red fluorescent Phalloidin Conjugate Working Solution for 45 min at room temperature in the dark. Afterwards, for 5 min, Hoechst 33258 was added to each well. The EVOS™ M5000 Imaging System (Thermo Fisher Scientific, Inc., Waltham, MA, USA) with a CMOS camera was employed for observing the cytoplasmic and nuclear modifications.

### 4.5. Statistical Analysis

The statistical analysis was performed using a t-test and one-way ANOVA followed by Dunnett’s post hoc test using GraphPad Prism version 6.0.0 (GraphPad Software, San Diego, CA, USA). The IC_50_ values were calculated using the same software. The differences between the groups were considered statistically significant if *p* < 0.05, as follows: * *p* < 0.05, ** *p* < 0.01, and *** *p* < 0.001. The graphs were generated using Python 3.11 (Python Software Foundation), using the Pandas submodule. 

### 4.6. HET-CAM Assay 

The assessment of the safety profile of BA esters and their liposomal formulations against living tissues was performed using the in ovo chorioallantoic membrane of the chick embryo. The standard protocol relied on the usage of a developing chorioallantoic membrane within an embryonated chicken (*Gallus domesticus*) egg. According to a modified version of the standard HET-CAM procedure [73], the standard procedures for incubations (37 °C and 50% relative humidity) were used for the tested eggs. A total of 4–5 mL of albumen was extracted during the third incubation day in order to detach the chorioallantoic membrane from the inner eggshell. A total of 600 μL of each sample (100 μM) were prepared in 0.5% DMSO and added on top of the developing CAM on day 10 of incubation for assessing their irritative potential. We employed a reaction–time method of prediction for the irritation potential [74]. The possible alterations of the vascular plexus of the treated CAMs were monitored for 300 s and the first appearance of hemorrhage, lysis, and vascular plexus coagulability were noted. Observation was performed through the use of stereomicroscopy (Discovery 8 Stereomicroscope by Zeiss, Jena, Germany) and images were captured using the Axio CAM 105 color camera (Jena, Germany), both before and 5 min after applying the test solutions. The obtained results were quantified as irritation factor (IF) values using the following formula:

$$\mathrm{IF}=5\;\times \;\frac{301-Sec\;H}{300}+7\;\times \;\frac{301-Sec\;L}{300}+9\;\times \;\frac{301-Sec\;C}{300}$$

where IF = irritation factor; *H* = hemorrhage; *L* = vascular lysis; *C* = coagulation; Sec *H* = first appearance of hemorrhage reactions (s); *Sec L* = first appearance of vessel lysis on CAM (s); *Sec C* = first appearance of coagulation. 

The obtained values were compared to a negative (distilled water) and a positive control (SLS 0.5%). The Luepke scale was used to assess the IF values with the following ranges: 0–0.9 for non-irritation, 1–4.9 for weak irritation, 5–8.9 for moderate irritation, and 9–21 for strong irritation [75].

### 4.7. Molecular Docking

Molecular docking was used to predict the binding poses and scores of the three BA esters as well as BA, to determine whether these chemical modifications improve the binding affinity of the parent compound against two of its targets, the anti-apoptotic proteins Bcl-XL and Bcl-2. This was a two-step process that involved ranking the four compounds using two separate docking softwares with different scoring functions. The 3D structures of Bcl-2, Bcl-XL, and NF-κB p50 were obtained from the RCSB Protein Data Bank [76], using the PDB entries 2YXJ (Bcl-XL), 4LVT (Bcl-2), and 1NFK (NF-κB p50). Firstly, the compounds were docked using PyRx’s Vina module [40,77]. To accomplish this, the protein file was optimized using the AutoDock Tools v1.5.6 suite (The Scripps Research Institute, La Jolla, CA, USA). Water molecules and the native co-crystallized ligand were removed, and Gasteiger charges were added to the protein. Structures of BA and its esters were sketched in Biovia Draw (Dasault Systems Biovia, San Diego, CA, USA) and then converted into 3D structures using PyRx v0.8’s Open Babel module (UFF force field). The docking grid box, which designates the docking space, was adjusted to fit the binding site where the native ligand resides for each protein, with the exception of 1NFK where the structure does not contain a small molecule inhibitor; therefore, the grid box was placed to include the RHD where the DNA is bound. 

In the second step, the compounds were re-docked into the same targets using Schrödinger’s Glide module [39,78,79]. For this purpose, the previously obtained 3D ligand structures of BA and its esters were prepared using Maestro’s LigPrep with the default features. Protein targets were prepared using Maestro’s Protein Preparation Wizard [80] with default parameters, which include multiple bond rectification, hydrogen atom addition and non-polar H merging, and clearance of water molecules beyond a 5 Å radius from the ligand. The wizard also ran a protein structure-processing step to identify and adjust errors and a protein–ligand complex refinement employing a successive restrained minimization with the featured OPLS_2005 force field. For docking space delimitation, the grid box was generated with Maestro’s receptor grid generation feature, which uses the native ligand as the central point to define the active site for the Bcl-2 and Bcl-XL binding site amino acid residues, to define the binding site for NF-κB p50 in the absence of a small molecule co-crystalized ligand. Subsequently, the grid box can be adjusted to better fit the active site. All docking experiments were validated by re-docking the native ligand into its active site (where possible) and computing the RMSD values of the docked and experimental poses, which did not exceed a 2 Å threshold. The compounds were ranked based on Vina’s and Glide’s docking scores. Ligand–protein binding features were interpreted using the best scored docking pose for each docked compound.

## 5. Conclusions 

Our study described the biological evaluation of a series of BA fatty esters and their respective liposomal formulations against breast (MCF-7), colon (HT-29), and lung (NCI-H460) cancer cells. Both the esters and their liposomes exerted good cytotoxic effects against the tested cancer cells in a dose- and time-dependent manner, some compounds even inhibiting cancer cells more potently compared to the positive control, 5-fluorouracil. But-BA-Lip proved the most effective cytotoxic compound, exhibiting lower IC_50_ values compared to its parent compound, BA, and its liposome, BA-Lip, against all tested cancer cells. The pro-apoptotic effect of the newly synthesized compounds might reside in their property to activate caspase-3/-7, leading to chromatin condensation and nuclear leakage in cancer cells. Furthermore, the in ovo HET-CAM assay has revealed that neither the BA’s fatty esters, nor their liposomal formulations exhibited any irritative effect on the chorioallantoic membrane, ensuring their safety in applications for local treatments. Future studies are warranted in order to establish the in vivo anticancer potential of BA’s fatty esters and their liposomes, leading to successful strategies for creating similar compounds with improved cytotoxic effects.

## Figures and Tables

**Table 1:** HT-29, MCF-7, NCI-H460 obtained IC_50_ values (μM) of BA, But-BA, Pal-BA, St-BA, BA-Lip, But-BA-Lip, Pal-BA-Lip, St-BA-Lip, and 5-FU after stimulation for 48 h.

Compound	MCF-7	HT-29	NCI-H460
5-FU	30.79 ± 0.34	82.53 ± 0.77	62.89 ± 0.28
BA	54.97 ± 0.87	91.16 ± 0.93	39.48 ± 0.74
BA-Lip	54.89 ± 0.26	59.04 ± 1.44	35.42 ± 0.91
Pal-BA	>100	>100	52.37 ± 0.35
Pal-BA-Lip	>100	70.06 ± 0.54	41.72 ± 0.94
St-BA	>100	>100	61.17 ± 1.48
St-BA-Lip	>100	>100	43.35 ± 1.06
But-BA	63.17 ± 0.28	77.72 ± 0.63	50.26 ± 0.53
But-BA-Lip	48.88 ± 1.32	30.57 ± 1.02	30.74 ± 1.16

**Table 2:** The effects of BA’s fatty esters and their liposomal formulations on cell viability 24 h and 48 h post stimulation with 100 μM.

	Viability (%)					
Compound (100 μM)	MCF-7		HT-29		NCI-H460	
	After 24 h	After 48 h	After 24 h	After 48 h	After 24 h	After 48 h
BA	45.44 ± 0.74	33.57 ± 1.85	58.05 ± 1.18	44.79 ± 1.12	40.97 ± 2.31	31.9 ± 0.74
BA-Lip	39.49 ± 1.16	32.48 ± 3.41	50.75 ± 0.96	37.4 ± 1.36	38.23 ± 0.46	25.15 ± 1.08
Pal-BA	71.62 ± 1.31	69.91 ± 1.8	69.62 ± 2.34	68.92 ± 2.09	44.62 ± 1.27	32.42 ± 0.51
Pal-BA-Lip	65.59 ± 0.82	63.86 ± 2.44	65.59 ± 0.87	41.69 ± 1.91	37.18 ± 2.34	26.08 ± 0.94
St-BA	74.72 ± 2.36	70.71 ± 0.84	66.54 ± 1.35	62.16 ± 1.07	50.84 ± 0.91	42.74 ± 1.74
St-BA-Lip	70.54 ± 0.97	66.81 ± 0.63	59.72 ± 0.76	51.7 ± 0.58	36.58 ± 3.45	27.48 ± 1.3
But-BA	68.59 ± 1.22	40.53 ± 2.19	61.59 ± 0.43	40.77 ± 0.22	49.45 ± 1.15	38.35 ± 1.33
But-BA-Lip	37.23 ± 0.88	30.76 ± 6.21	52.83 ± 0.56	30.77 ± 1.29	29.97 ± 0.69	17.8 ± 2.61

**Table 3:** The irritation factors for BA, BA-Lip, But-BA, But-BA-Lip, Pal-BA, Pal-BA-Lip, St-BA, St-BA-Lip, and Lip.

Sample	IF	Type of Effect
H_2_O	0	No irritation
SDS	17.19 ± 0.16	Severe irritation
DMSO	0	No irritation
Lip	0	No irritation
BA	0	No irritation
Ba-Lip	0	No irritation
Pal-BA	0	No irritation
Pal-BA-Lip	0	No irritation
St-BA	0	No irritation
St-BA-Lip	0	No irritation
But-BA	0	No irritation
But-BA-Lip	0	No irritation

**Table 4:** Calculated docking scores of BA and its fatty acid esters against Bcl-2 and Bcl-XL.

Ligand	Bcl-2 (PDB ID: 4LVT)		Bcl-XL (PDB ID: 2YXJ)		NF-κB (PDB ID: 1NFK)	
	Vina Docking Score	Glide Docking Score	Vina Docking Score	Glide Docking Score	Vina Docking Score	Glide Docking Score
BA	−7.7	−4.3	−8.5	−4.9	−8	−3.3
But-BA	−7.8	−3.8	−8.4	−3.9	−7.6	−4.1
Pal-BA	−8.5	−6.4	−9.1	−6.5	−6	−5.1
St-BA	−7.6	−6.1	−8.7	−6.7	−5.1	−4.7

## Data Availability

The original contributions presented in the study are included in the article, further inquiries can be directed to the corresponding author.

## References

[1] Gullett N.P., Ruhul Amin A.R.M., Bayraktar S., Pezzuto J.M., Shin D.M., Khuri F.R., Aggarwal B.B., Surh Y.-J., Kucuk O. (2010). Cancer Prevention with Natural Compounds. Semin. Oncol..

[2] Bray F., Laversanne M., Sung H., Ferlay J., Siegel R.L., Soerjomataram I., Jemal A. (2024). Global Cancer Statistics 2022: GLOBOCAN Estimates of Incidence and Mortality Worldwide for 36 Cancers in 185 Countries. CA. Cancer J. Clin..

[3] Greenman C., Stephens P., Smith R., Dalgliesh G.L., Hunter C., Bignell G., Davies H., Teague J., Butler A., Stevens C. (2007). Patterns of Somatic Mutation in Human Cancer Genomes. Nature.

[4] Chaib S., López-Domínguez J.A., Lalinde-Gutiérrez M., Prats N., Marin I., Boix O., García-Garijo A., Meyer K., Muñoz M.I., Aguilera M. (2024). The Efficacy of Chemotherapy Is Limited by Intratumoral Senescent Cells Expressing PD-L2. Nat. Cancer.

[5] Yahya E.B., Alqadhi A.M. (2021). Recent Trends in Cancer Therapy: A Review on the Current State of Gene Delivery. Life Sci..

[6] Huang M., Lu J.-J., Ding J. (2021). Natural Products in Cancer Therapy: Past, Present and Future. Nat. Products Bioprospect..

[7] Lin S., Chang C., Hsu C., Tsai M., Cheng H., Leong M.K., Sung P., Chen J., Weng C. (2020). Natural Compounds as Potential Adjuvants to Cancer Therapy: Preclinical Evidence. Br. J. Pharmacol..

[8] Chudzik M., Korzonek-Szlacheta I., Król W. (2015). Triterpenes as Potentially Cytotoxic Compounds. Molecules.

[9] Elmore S. (2007). Apoptosis: A Review of Programmed Cell Death. Toxicol. Pathol..

[10] Gilmore T.D. (2006). Introduction to NF-ΚB: Players, Pathways, Perspectives. Oncogene.

[11] Hordyjewska A., Ostapiuk A., Horecka A., Kurzepa J. (2019). Betulin and Betulinic Acid: Triterpenoids Derivatives with a Powerful Biological Potential. Phytochem. Rev..

[12] Pisha E., Chai H., Lee I.-S., Chagwedera T.E., Farnsworth N.R., Cordell G.A., Beecher C.W.W., Fong H.H.S., Kinghorn A.D., Brown D.M. (1995). Discovery of Betulinic Acid as a Selective Inhibitor of Human Melanoma That Functions by Induction of Apoptosis. Nat. Med..

[13] Ghiulai R., Mioc M., Racoviceanu R., Prodea A., Milan A., Coricovac D., Dehelean C., Avram Ș., Zamfir A.D., Munteanu C.V.A. (2022). Structural Investigation of Betulinic Acid Plasma Metabolites by Tandem Mass Spectrometry. Molecules.

[14] Lou H., Li H., Zhang S., Lu H., Chen Q. (2021). A Review on Preparation of Betulinic Acid and Its Biological Activities. Molecules.

[15] Adepoju F.O., Duru K.C., Li E., Kovaleva E.G., Tsurkan M.V. (2023). Pharmacological Potential of Betulin as a Multitarget Compound. Biomolecules.

[16] Hsu T.-I., Wang M.-C., Chen S.-Y., Huang S.-T., Yeh Y.-M., Su W.-C., Chang W.-C., Hung J.-J. (2012). Betulinic Acid Decreases Specificity Protein 1 (Sp1) Level via Increasing the Sumoylation of Sp1 to Inhibit Lung Cancer Growth. Mol. Pharmacol..

[17] Rzeski W., Stepulak A., Szymański M., Sifringer M., Kaczor J., Wejksza K., Zdzisińska B., Kandefer-Szerszeń M. (2006). Betulinic Acid Decreases Expression of Bcl-2 and Cyclin D1, Inhibits Proliferation, Migration and Induces Apoptosis in Cancer Cells. Naunyn. Schmiedebergs. Arch. Pharmacol..

[18] Guo Y., Zhu H., Weng M., Wang C., Sun L. (2020). Chemopreventive Effect of Betulinic Acid via MTOR -Caspases/Bcl2/Bax Apoptotic Signaling in Pancreatic Cancer. BMC Complement. Med. Ther..

[19] Zhong Y., Liang N., Liu Y., Cheng M.-S. (2021). Recent Progress on Betulinic Acid and Its Derivatives as Antitumor Agents: A Mini Review. Chin. J. Nat. Med..

[20] Soica C., Danciu C., Savoiu-Balint G., Borcan F., Ambrus R., Zupko I., Bojin F., Coricovac D., Ciurlea S., Avram S. (2014). Betulinic Acid in Complex with a Gamma-Cyclodextrin Derivative Decreases Proliferation and in Vivo Tumor Development of Non-Metastatic and Metastatic B164A5 Cells. Int. J. Mol. Sci..

[21] Nistor G., Trandafirescu C., Prodea A., Milan A., Cristea A., Ghiulai R., Racoviceanu R., Mioc A., Mioc M., Ivan V. (2022). Semisynthetic Derivatives of Pentacyclic Triterpenes Bearing Heterocyclic Moieties with Therapeutic Potential. Molecules.

[22] Saneja A., Arora D., Kumar R., Dubey R.D., Panda A.K., Gupta P.N. (2018). Therapeutic Applications of Betulinic Acid Nanoformulations. Ann. N. Y. Acad. Sci..

[23] Huang C.B., Alimova Y., Myers T.M., Ebersole J.L. (2011). Short- and Medium-Chain Fatty Acids Exhibit Antimicrobial Activity for Oral Microorganisms. Arch. Oral Biol..

[24] Bravo-Santano N., Ellis J.K., Calle Y., Keun H.C., Behrends V., Letek M. (2019). Intracellular Staphylococcus Aureus Elicits the Production of Host Very Long-Chain Saturated Fatty Acids with Antimicrobial Activity. Metabolites.

[25] Liu S., Ruan W., Li J., Xu H., Wang J., Gao Y., Wang J. (2008). Biological Control of Phytopathogenic Fungi by Fatty Acids. Mycopathologia.

[26] Siena L., Cipollina C., Di Vincenzo S., Ferraro M., Bruno A., Gjomarkaj M., Pace E. (2018). Electrophilic Derivatives of Omega-3 Fatty Acids Counteract Lung Cancer Cell Growth. Cancer Chemother. Pharmacol..

[27] Morin C., Rousseau É., Fortin S. (2013). Anti-Proliferative Effects of a New Docosapentaenoic Acid Monoacylglyceride in Colorectal Carcinoma Cells. Prostaglandins, Leukot. Essent. Fat. Acids.

[28] Irby D., Du C., Li F. (2017). Lipid–Drug Conjugate for Enhancing Drug Delivery. Mol. Pharm..

[29] Jóźwiak M., Filipowska A., Fiorino F., Struga M. (2020). Anticancer Activities of Fatty Acids and Their Heterocyclic Derivatives. Eur. J. Pharmacol..

[30] Li F., Snow-Davis C., Du C., Bondarev M.L., Saulsbury M.D., Heyliger S.O. (2016). Preparation and Characterization of Lipophilic Doxorubicin Pro-Drug Micelles. J. Vis. Exp..

[31] El-Desouky S.K. (2023). A Cytotoxic Lupeol Fatty Acid Ester and Other Pentacyclic Triterpenes from Salvadora Persica Seeds. Nat. Prod. Sci..

[32] Milan A., Mioc M., Mioc A., Marangoci N., Racoviceanu R., Mardale G., Bălan-Porcărașu M., Rotunjanu S., Şoica I., Șoica C. (2024). Exploring the Antimelanoma Potential of Betulinic Acid Esters and Their Liposomal Nanoformulations. Processes.

[33] Luepke N.P., Kemper F.H. (1986). The HET-CAM Test: An Alternative to the Draize Eye Test. Food Chem. Toxicol..

[34] Anaya-Eugenio G.D., Eggers N.A., Ren Y., Rivera-Chávez J., Kinghorn A.D., Carcache De Blanco E.J. (2020). Apoptosis Induced by (+)-Betulin Through NF-ΚB Inhibition in MDA-MB-231 Breast Cancer Cells. Anticancer Res..

[35] Mioc M., Mioc A., Prodea A., Milan A., Balan-Porcarasu M., Racoviceanu R., Ghiulai R., Iovanescu G., Macasoi I., Draghici G. (2022). Novel Triterpenic Acid—Benzotriazole Esters Act as Pro-Apoptotic Antimelanoma Agents. Int. J. Mol. Sci..

[36] Kazakova O., Mioc A., Smirnova I., Baikova I., Voicu A., Vlaia L., Macașoi I., Mioc M., Drăghici G., Avram Ş. (2021). Novel Synthesized N-Ethyl-Piperazinyl-Amides of C2-Substituted Oleanonic and Ursonic Acids Exhibit Cytotoxic Effects through Apoptotic Cell Death Regulation. Int. J. Mol. Sci..

[37] Kazakova O., Șoica C., Babaev M., Petrova A., Khusnutdinova E., Poptsov A., Macașoi I., Drăghici G., Avram Ș., Vlaia L. (2021). 3-Pyridinylidene Derivatives of Chemically Modified Lupane and Ursane Triterpenes as Promising Anticancer Agents by Targeting Apoptosis. Int. J. Mol. Sci..

[38] Meira C.S., do Espírito Santo R.F., dos Santos T.B., Orge I.D., Silva D.K.C., Guimarães E.T., de Aragão França L.S., Barbosa-Filho J.M., Moreira D.R.M., Soares M.B.P. (2017). Betulinic Acid Derivative BA5, a Dual NF-KB/Calcineurin Inhibitor, Alleviates Experimental Shock and Delayed Hypersensitivity. Eur. J. Pharmacol..

[39] Friesner R.A., Banks J.L., Murphy R.B., Halgren T.A., Klicic J.J., Mainz D.T., Repasky M.P., Knoll E.H., Shelley M., Perry J.K. (2004). Glide: A New Approach for Rapid, Accurate Docking and Scoring. 1. Method and Assessment of Docking Accuracy. J. Med. Chem..

[40] Trott O., Olson A.J. (2010). AutoDock Vina: Improving the Speed and Accuracy of Docking with a New Scoring Function, Efficient Optimization, and Multithreading. J. Comput. Chem..

[41] Wang H., Guo M., Wei H., Chen Y. (2023). Structural Basis of the Specificity and Interaction Mechanism of Bmf Binding to Pro-Survival Bcl-2 Family Proteins. Comput. Struct. Biotechnol. J..

[42] Souers A.J., Leverson J.D., Boghaert E.R., Ackler S.L., Catron N.D., Chen J., Dayton B.D., Ding H., Enschede S.H., Fairbrother W.J. (2013). ABT-199, a Potent and Selective BCL-2 Inhibitor, Achieves Antitumor Activity While Sparing Platelets. Nat. Med..

[43] Lee E.F., Czabotar P.E., Smith B.J., Deshayes K., Zobel K., Colman P.M., Fairlie W.D. (2007). Crystal Structure of ABT-737 Complexed with Bcl-XL: Implications for Selectivity of Antagonists of the Bcl-2 Family. Cell Death Differ..

[44] Fulda S. (2009). Betulinic Acid: A Natural Product with Anticancer Activity. Mol. Nutr. Food Res..

[45] Thurnher D., Turhani D., Pelzmann M., Wannemacher B., Knerer B., Formanek M., Wacheck V., Selzer E. (2003). Betulinic Acid: A New Cytotoxic Compound against Malignant Head and Neck Cancer Cells. Head Neck.

[46] Zuco V., Supino R., Righetti S.C., Cleris L., Marchesi E., Gambacorti-Passerini C., Formelli F. (2002). Selective Cytotoxicity of Betulinic Acid on Tumor Cell Lines, but Not on Normal Cells. Cancer Lett..

[47] Fulda S., Jeremias I., Steiner H.H., Pietsch T., Debatin K.-M. (1999). Betulinic Acid: A New Cytotoxic Agent against Malignant Brain-Tumor Cells. Int. J. Cancer.

[48] Venepally V., Reddy Jala R.C. (2017). An Insight into the Biological Activities of Heterocyclic–Fatty Acid Hybrid Molecules. Eur. J. Med. Chem..

[49] Viklund F., Alander J., Hult K. (2003). Antioxidative Properties and Enzymatic Synthesis of Ascorbyl FA Esters. J. Am. Oil Chem. Soc..

[50] Mustafa J., Khan S.I., Ma G., Walker L.A., Khan I.A. (2004). Synthesis and Anticancer Activities of Fatty Acid Analogs of Podophyllotoxin. Lipids.

[51] Pinzaru I., Trandafirescu C., Szabadai Z., Mioc M., Ledeti I., Coricovac D., Ciurlea S., Ghiulai R.M., Crainiceanu Z., Simu G. (2014). Synthesis and Biological Evaluation of Some Pentacyclic Lupane Triterpenoid Esters. Rev. Chim..

[52] Liu Y., Gao D., Zhang X., Liu Z., Dai K., Ji B., Wang Q., Luo L. (2016). Antitumor Drug Effect of Betulinic Acid Mediated by Polyethylene Glycol Modified Liposomes. Mater. Sci. Eng. C.

[53] Suresh C., Zhao H., Gumbs A., Chetty C.S., Bose H.S. (2012). New Ionic Derivatives of Betulinic Acid as Highly Potent Anti-Cancer Agents. Bioorg. Med. Chem. Lett..

[54] Kvasnica M., Sarek J., Klinotova E., Dzubak P., Hajduch M. (2005). Synthesis of Phthalates of Betulinic Acid and Betulin with Cytotoxic Activity. Bioorg. Med. Chem..

[55] Kessler J.H., Mullauer F.B., de Roo G.M., Medema J.P. (2007). Broad in Vitro Efficacy of Plant-Derived Betulinic Acid against Cell Lines Derived from the Most Prevalent Human Cancer Types. Cancer Lett..

[56] Shu Q., Wu J., Chen Q. (2019). Synthesis, Characterization of Liposomes Modified with Biosurfactant MEL-A Loading Betulinic Acid and Its Anticancer Effect in HepG2 Cell. Molecules.

[57] Fulda S. (2008). Betulinic Acid for Cancer Treatment and Prevention. Int. J. Mol. Sci..

[58] Eskandari E., Eaves C.J. (2022). Paradoxical Roles of Caspase-3 in Regulating Cell Survival, Proliferation, and Tumorigenesis. J. Cell Biol..

[59] Yaozu Z., Liu Y., Zhao H., Peng P., Tingbao Z., Jincao C. (2021). Betulinic Acid Inhibits Glioma Cell Viability by Downregulation of NF-ΚB and Enhancement of Apoptosis. Trop. J. Pharm. Res..

[60] Chen F., Zhong Z., Tan H.Y., Guo W., Zhang C., Cheng C., Wang N., Ren J., Feng Y. (2020). Suppression of LncRNA MALAT1 by Betulinic Acid Inhibits Hepatocellular Carcinoma Progression by Targeting IAPs via MiR-22-3p. Clin. Transl. Med..

[61] Kutkowska J., Strzadala L., Rapak A. (2021). Hypoxia Increases the Apoptotic Response to Betulinic Acid and Betulin in Human Non-Small Cell Lung Cancer Cells. Chem. Biol. Interact..

[62] Shen M., Hu Y., Yang Y., Wang L., Yang X., Wang B., Huang M. (2019). Betulinic Acid Induces ROS-Dependent Apoptosis and S-Phase Arrest by Inhibiting the NF- κ B Pathway in Human Multiple Myeloma. Oxid. Med. Cell. Longev..

[63] Rachek L.I., Musiyenko S.I., LeDoux S.P., Wilson G.L. (2007). Palmitate Induced Mitochondrial Deoxyribonucleic Acid Damage and Apoptosis in L6 Rat Skeletal Muscle Cells. Endocrinology.

[64] Buratta M., Castigli E., Sciaccaluga M., Pellegrino R.M., Spinozzi F., Roberti R., Corazzi L. (2008). Loss of Cardiolipin in Palmitate-treated GL15 Glioblastoma Cells Favors Cytochrome c Release from Mitochondria Leading to Apoptosis. J. Neurochem..

[65] Semaan J., El-Hakim S., Ibrahim J.-N., Safi R., Elnar A.A., El Boustany C. (2020). Comparative Effect of Sodium Butyrate and Sodium Propionate on Proliferation, Cell Cycle and Apoptosis in Human Breast Cancer Cells MCF-7. Breast Cancer.

[66] Chopin V., Toillon R., Jouy N., Bourhis X. (2002). Le Sodium Butyrate Induces P53-independent, Fas-mediated Apoptosis in MCF-7 Human Breast Cancer Cells. Br. J. Pharmacol..

[67] Wang L., Luo H.-S., Xia H. (2009). Sodium Butyrate Induces Human Colon Carcinoma HT-29 Cell Apoptosis through a Mitochondrial Pathway. J. Int. Med. Res..

[68] Quagliariello V., Masarone M., Armenia E., Giudice A., Barbarisi M., Caraglia M., Barbarisi A., Persico M. (2018). Chitosan-Coated Liposomes Loaded with Butyric Acid Demonstrate Anticancer and Anti-Inflammatory Activity in Human Hepatoma HepG2 Cells. Oncol. Rep..

[69] Evans L.M., Cowey S.L., Siegal G.P., Hardy R.W. (2009). Stearate Preferentially Induces Apoptosis in Human Breast Cancer Cells. Nutr. Cancer.

[70] Wilson T.., Steck W. (2000). A Modified HET–CAM Assay Approach to the Assessment of Anti-Irritant Properties of Plant Extracts. Food Chem. Toxicol..

[71] Rednic R., Macasoi I., Pinzaru I., Dehelean C.A., Tomescu M.-C., Susan M., Feier H. (2022). Pharmaco-Toxicological Assessment of the Combined Cytotoxic Effects of Digoxin and Betulinic Acid in Melanoma Cells. Life.

[72] Ghiulai R., Mioc A., Racoviceanu R., Mioc M., Milan A., Prodea A., Semenescu A., Dehelean C., Barbu Tudoran L., Avram Ș. (2022). The Anti-Melanoma Effect of Betulinic Acid Functionalized Gold Nanoparticles: A Mechanistic In Vitro Approach. Pharmaceuticals.

[73] Maghiari A.L., Coricovac D., Pinzaru I.A., Macașoi I.G., Marcovici I., Simu S., Navolan D., Dehelean C. (2020). High Concentrations of Aspartame Induce Pro-Angiogenic Effects in Ovo and Cytotoxic Effects in HT-29 Human Colorectal Carcinoma Cells. Nutrients.

[74] Wang F., Zhang C., Wang B. (2021). Application of in Vitro Methods to Evaluate the Safety of Baby Care Products. Toxicol. Vitr..

[75] Luepke N.P. (1985). Hen’s Egg Chorioallantoic Membrane Test for Irritation Potential. Food Chem. Toxicol..

[76] Berman H.M. (2000). The Protein Data Bank. Nucleic Acids Res..

[77] Dallakyan S., Olson A.J. (2015). Small-Molecule Library Screening by Docking with PyRx. Chemical Biology: Methods and Protocols.

[78] Friesner R.A., Murphy R.B., Repasky M.P., Frye L.L., Greenwood J.R., Halgren T.A., Sanschagrin P.C., Mainz D.T. (2006). Extra Precision Glide: Docking and Scoring Incorporating a Model of Hydrophobic Enclosure for Protein−Ligand Complexes. J. Med. Chem..

[79] Halgren T.A., Murphy R.B., Friesner R.A., Beard H.S., Frye L.L., Pollard W.T., Banks J.L. (2004). Glide: A New Approach for Rapid, Accurate Docking and Scoring. 2. Enrichment Factors in Database Screening. J. Med. Chem..

[80] Madhavi Sastry G., Adzhigirey M., Day T., Annabhimoju R., Sherman W. (2013). Protein and Ligand Preparation: Parameters, Protocols, and Influence on Virtual Screening Enrichments. J. Comput. Aided. Mol. Des..

